# Death of Prof. George Hadley, M. D.

**Published:** 1877-11

**Authors:** 


### PROF. GEORGE HADLEY, M. D.

ACTION OF THE ERIE COUNTY MEDICAL SOCIETY UPON HIS DEATH—MEMORIAL ADOPTED.

Pursuant to call a meeting of the Erie County Medical Society was held Wednesday evening, October 17th, 1877, at the college building, corner Main and Virginia streets, for the purpose of taking action on the death of Prof. George Hadley. The attendance was large. Dr. Edward Storck, vice-president of the association, presided, and Dr. D. W. Harrington discharged the duties of secretary.

Upon calling the meeting to order Dr. Storck addressed his colleagues as follows:

*Gentlemen of Erie County Medical Society:*

Again it has become my duty, in the absence of the President, to call a meeting of this society on account of the death of our collegue, one of the oldest, most honorable and most distinguished members of our society, Prof. Hadley, who for many years has held the chair of chemistry in the Buffalo Medical College so honorably, has so distinguished himself as an expert that he gained a national reputation. Prof. Hadley's name was known not only here, but, I may add, all over the state; yes, all over the country. He died at his residence yesterday morning. We mourn in him a great and honored member, a distinguished member of the profession, a teacher whose place can hardly be filled, and it is highly proper that this society should take proper action on the death of our departed colleague, and to give expression to our feeling of sympathy with the family and express our feeling of honor and respect for our departed colleague.

Dr. Thomas F. Rochester in next moving for the appointment of a committee of three to prepare a memorial expressive of the sentiments of the society, said:

*Mr. President and Gentlemen:*

Perhaps it is proper that I should say a few words on this very sad occasion, from the fact that my acquaintance with Dr. Hadley probably antedates that of any other person in this city. I have known Dr. Hadley intimately and well for over twenty-five years; known him as a man, as a chemist, as a friend for that whole period and for twenty-five years as a colleague, and my relations with him have not only been intimate in that respect, but I have also had the privilege of being his medical adviser during that long period, not only of himself, but his honored father and mother. It was my sad duty to be with them in their last illnesses and also in that of his sister, and therefore you see that my relation has been extremely intimate and that I have a personal quite as much as a professional loss to deplore. It seems hardly possible to express the feelings which I believe to pervade the hearts of all the professional men—the medical men in this city, with regard to the loss that we have sustained. Every physician feels that he has lost in Prof. Hadley an associate, a friend, a co-laborer whom it was an honor to know, and to claim as a member of the common profession. You, Mr. President, have so fully and so well expressed the feeling that is entertained by the profession of this city, that it is unnecessary for me to say anything more on the subject. As one of the colleagues of this institution we feel indeed as you have well said, that we have lost one whose position can scarcely be filled, that it will be difficult indeed to find one to occupy his place.

At the conclusion of Dr. Rochester's remarks the chair named a committee to prepare the memorial.

The committee retired, and during their absence Dr. Julius F. Miner addressed the society as follows.

I am not prepared on this occasion to pronounce a eulogy on Prof. Hadley. His whole life is his eulogy. His reputation with the profession in this vicinity is the best eulogy that could possibly be made upon the life and character of the man with as clear a head and as pure a heart as is ever given to mortal. Dr. Hadley has lived the most circumspect and exemplary professional life of any man almost that we have among our number, that we have ever known; and he is endeared to all the members of the profession. The old men of the profession now are his boys. The advanced men of the profession look back to the time when he was their teacher, and he was always their friend. I think there is no physician in the vicinity, in speaking of Dr. Hadley, but what he would say he was one of their best advisers. As his colleague, I can only say that we have always regarded Dr. Hadley's conclusions as sufficient authority for any course of action. His arguments were always clear and satisfactory and his conclusions irresistable, and in every matter which either agitated us in any professional matter, or in any college matter, Prof. Hadley has been looked upon as authority. If I knew what Prof. Hadley thought was proper and right on any matter, I knew perfectly well what I ought to think was proper and right; and that I did think. He was infallible in his judgment, he was kind in his nature, he was benevolent, he was not selfish. He could not be liberal so far as munificence in the affairs of the society or the profession or the college were concerned, in any great degree. He was too pure a minded and too honest a man to be able to do any very great or ostentatious act of benevolence or charity, but he was pure in his purposes, he was correct in everything which he undertook, and I have never known so pure a man whose perceptions of right and wrong were so clear, and who was so wholly governed by what his convictions of right might be, as Prof. Hadley. He was really remarkable, too, in his adherence to and his love for the truth. But I am not at liberty to occupy your attention any longer.

The committee upon their return presented the following

MEMORIAL:

After a long, earnest, industrious and truthful life George Hadley has passed from this busy and tumultuous world into that eternal rest which is the ultimate destination of all good men—for to this class he certainly belonged. He died on October 16th, 1877, at the age of 64 years, 3 months and 26 days—not old but of ripe years. He preserved to the last his clear mind and discriminating intellect. He knew that his end was near at hand, and calmly, quietly and characteristically looked for and met it. He was emphatically a persistent and discerning worker in the paths of science. His mind was strictly searching and analytical; he sought for fact and demonstration, and never allowed himself to be misled by conjecture, with such natural, and it may be added inherited mental proclivities, it would have been singular indeed if he had not attained great distinction in chemistry, the branch of art to which he had most conscientiously devoted his life. It is probably not an extravagant statement to say that his superior as a medical and analytical chemist never existed. His patience, perseverance, ingenuity and precise accuracy, based upon a most intimate knowledge of chemical relations, gave to his opinions and decisions a weight and reliance which carried conviction, and from which there was almost never an appeal. Besides his eminence in his proper profession—Chemistry—he was well versed in Mineralogy, Botany, Conchology and other kindred departments of the Natural Sciences. With all these accomplishments he was modest and retiring almost to diffidence. His feeling toward all humanity was kindly. His heart was almost womanly in its gentle tenderness. Yet his convictions were strong, and he always was firm and unwavering in the maintenance of his opinions. In the professions of medicine and of law he was widely and most favorably known, in the latter from his distinction as an expert in medico-chemical legal investigations. Popularly he was much less prominent than many of much less excellence. Simply because he shrank from notoriety and publicity. This man whose individual excellence and integrity were the basis on which his talents were built and rested we shall see no more. We rejoice that he was so long spared as an example for us to emulate. We are glad that our professional lot was cast with his for so extended a period. We are proud that our associate has contributed much to elevate and ennoble science. We congratulate his bereaved family on the honorable name and distinction he has left to them as the most precious of legacies.

On motion of Dr. Loomis the report of the committee was accepted and adopted.

On motion of Dr. Trowbridge, it was resolved that the condolence of the society be tendered the family; also that the members attend the funeral of Prof. Hadley in a body.

Brief addresses were also made by Dr. Charles W. Harvey, Dr. Henry Nichell, Dr. H. N. Loomis, Dr. E. N. Brush, Dr. John S. Trowbridge, Dr. Bartlett and Dr. W. W. Miner, after which the meeting adjourned.

### THE STUDENTS' MEMORIAL.

At a meeting of the students of the Medical College, to take action with reference to the death of Prof. George Hadley, the following memorial was adopted.

MEDICAL COLLEGE, BUFFALO, Oct. 17, 1877.

We, as students having been called upon to mourn the death of our esteemed and beloved teacher, Prof. George Hadley, deeply deplore our loss. While we are conscious that no words of ours can add to the high estimation in which the deceased was held by all who knew him, whether as a scientific scholar, a patient and pains-taking teacher or as a genial, kindly gentleman; and while we know his high intellectual attainments and spotless character, will receive at the hands of others a far more fitting tribute than we can give, still we deem it not inappropriate to speak of our relations with him who was our teacher, and whom we have known only to respect and love. Gentle and amiable in his disposition, he was firm in principle and purpose, and his statements and opinions were always truthful and reliable. He was pre-eminently the student's friend, ever ready to instruct, advise and assist, and when he felt that his teachings were understood his face would light up with a glow of satisfied pride, which plainly told us how unselfishly he was working for our good. Showing toward all the same untiring patience, he could not fail to gain the esteem and love of all. In his death, the college with which he was so long connected, the cause of science to which his life was devoted, and the city in which he lived, have sustained an irreparable loss. Especially to his bereaved family would we tender our sincere and heartfelt sympathy; but while we join with them in lamenting their loss we would not grieve as those who are without hope, for of one who lived so sweet, so pure and gentle a life as his, it may be truly said it was but a single step from earth to heaven.

On behalf of the students,

EARNEST WENDE,
J. H. WRIGHT,
CHAS. L. VAN PELT,
D. E. FULLER,
CHAS. A. WALL.
Committee.

